# Deep hydration of an Li_7–3*x*_La_3_Zr_2_ *M* ^III^_*x*_O_12_ solid-state electrolyte material: a case study on Al- and Ga-stabilized LLZO

**DOI:** 10.1107/S2053229621012250

**Published:** 2021-12-06

**Authors:** Günther J. Redhammer, Gerold Tippelt, Daniel Rettenwander

**Affiliations:** a Chemistry and Physics of Materials, University of Salzburg, Jakob Haringerstrasse 2A, 5020 Salzburg, Austria; b Department of Materials Science and Engineering, NTNU Norwegian University of Science and Technology, Trondheim, Norway

**Keywords:** solid-state electrolyte, LLZO, hydro­thermal degradation, crystal structure, structure analysis, garnet, lattice expansion

## Abstract

Single crystals of an Li-stuffed, Al- and Ga-stabilized garnet-type solid-state electrolyte material, Li_7_La_3_Zr_2_O_12_ (LLZO), have been analysed using single-crystal X-ray diffraction to determine the pristine structural state immediately after synthesis *via* ceramic sinter­ing techniques. Hydro­thermal treatment at 150 °C for 28 d induces a phase transition in the Al-stabilized com­pound from the commonly observed cubic *Ia*  *d* structure to the acentric *I*  3*d* subtype. Li^I^ ions at the inter­stitial octa­hedrally (4 + 2-fold) coordinated 48*e* site are most easily extracted and Al^III^ ions order onto the tetra­hedral 12*a* site. Deep hydration induces a distinct depletion of Li^I^ at this site, while the second tetra­hedral site, 12*b*, suffers only minor Li^I^ loss. Charge balance is maintained by the incorporation of H^I^, which is bonded to an O atom. Hydration of Ga-stabilized LLZO induces similar effects, with com­plete depletion of Li^I^ at the 48*e* site. The Li^I^/H^I^ exchange not only leads to a distinct increase in the unit-cell size, but also alters some bonding topology, which is discussed here.

## Abstract 2

Hydro­thermally treated Al- and Ga-stabilized stuffed Li garnets, nominally Li_7_La_3_Zr_2_O_12_, have been investigated by single-crystal X-ray diffraction to determine structural and site-occupation alterations due to Li^I^/H^I^ exchange.

## Introduction

The garnet family, *X*_3_ *Y*_2_ *Z*_3_O_12_, has been well described mineralogically and crystallographically in recent decades (Novak & Gibbs, 1971 ▸), and is of inter­est to a range of scientists from the fields of geoscience and technology, due to its thermodynamic stability in a variety of geological environments and its flexible structure, which can host ∼60 different chemical elements as major and minor components (Geiger, 2013 ▸; Baxter *et al.*, 2013 ▸). Furthermore, the so-called Li-stuffed garnets, *e.g.* Li_4_La_3_Zr_2_Li_3_O_12_, or as sum formula, Li_7_La_3_Zr_2_O_12_ (LLZO), have raised particular inter­est as promising materials for use as solid-state electrolytes in all solid-state Li batteries due to their superior Li-ion conductivity (Cussen, 2010 ▸; Wang *et al.*, 2020 ▸; Murugan *et al.*, 2007 ▸; Samson *et al.*, 2019 ▸). Pure end-member LLZO is tetra­gonal, has the space group *I*4_1_/*acd* (Awaka *et al.*, 2009 ▸) and has distinctly lower Li-ion conductivities. The high Li-ion conductivity is associated with the ‘standard’ cubic garnet structure with *Ia* 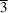 *d* symmetry. The latter can be stabilized by various aliovalent substitutions, *e.g.* by small amounts of Al^III^, which – in the first experiments – entered the structure as a contaminant from the corundum crucibles during synthesis (Geiger *et al.*, 2011 ▸; Buschmann *et al.*, 2011 ▸). The incorporation of Ga^III^ into LLZO also increases the Li-ion conductivity, but induces a reduction of the sym­metry to *I* 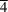 3*d* (Rettenwander *et al.*, 2016 ▸; Wagner *et al.*, 2016*a* ▸; Robben *et al.*, 2016 ▸), *i.e.* the space group of hydro­garnet Ca_3_Al_2_(O_4_H_4_)_3_ (Lager *et al.*, 1987 ▸). A stabilization of the cubic structure for nominally pure LLZO is also possible by the uptake of H^I^, which raises a question about LLZO stability in hydrous environments (Larraz *et al.*, 2013 ▸). Several studies have investigated the role of Li^I^/H^I^ exchange under different environmental conditions and found that LLZO-type materials are distinctly unstable in the presence of moisture (Ma *et al.*, 2015 ▸; Galven *et al.*, 2011 ▸, 2012 ▸, 2013 ▸; Larraz *et al.*, 2015 ▸; Orera *et al.*, 2016 ▸; Liu *et al.*, 2019 ▸). Surfaces quickly degrade with the formation of LiOH and Li_2_CO_3_, and an increase in unit-cell parameters is observed as Li^I^/H^I^ exchange progresses. Until now, only a few studies have systematically investigated the mechanisms behind this structural degradation. In recent studies, we have shown, using diffraction methods, that significant Li^I^ is especially lost from the inter­stitial sites of the structure in Al^III^-, Ga^III^- and Ta^V^-substituted LLZOs during ageing at room temperature under high humidity (Hiebl *et al.*, 2019 ▸; Redhammer *et al.*, 2021*a* ▸,*b* ▸). In this article, we report on the deep hydration of Al- and Ga-substituted LLZO using hydro­thermal treatment of single-crystalline material.

## Experimental methodology

### Synthesis and aging of material

Single crystals of Al- and Ga-stabilized LLZO were obtained using a solid-state ceramic sinter­ing method, which has been described in detail elsewhere (Rettenwander *et al.*, 2016 ▸; Wagner *et al.*, 2016*a* ▸). In brief, Li_2_CO_3_ (with an excess of 10%), La_2_O_3_, ZrO_2_ and Al_2_O_3_ or Ga_2_O_3_ were carefully mixed in the required stoichiometric proportions for the nominal com­positions Li_6.55_La_3_Zr_2_Al_0.15_O_12_ (Al15-LLZO) and Li_5.8_La_3_Zr_2_Ga_0.4_O_12_ (Ga40-LLZO). The mixtures were pressed into pellets and preheated at 850 °C for 4 h for deca­rbonatization. The samples were subsequently milled in a high-energy ball mill under alcohol using ZrO_2_ balls, then dried, pelletized and sintered at 1230 °C for 6 h. This yielded a dense ceramic consisting of large individual crystallites of up to 150 µm. Structural refinements were carried out on the fresh LLZOs within 24 h of synthesis to assess the unaltered structural state. Parts of the pellets were crushed carefully and aged in a 45 ml Teflon-lined autoclave (25 mg sample and 250 ml distilled water) at 150 °C for a period of 28 d. The pH value of the liquid, in which the crystals were submerged, was measured after the experiment; the pH value was ∼13 for both com­positions. The hydrated single LLZO crystallites were then filtered off and the remaining liquid was left to evaporate. The resulting precipitate was identified as Li_2_CO_3_ using powder diffraction (PXRD), *i.e.* proving that Li^I^ was extracted from the LLZO material.

### Refinement

Information on data collection and refinement results is given in Table 1 ▸. Indexing the diffraction data for pristine Al-LLZO yields the space-group symmetry *Ia* 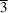 *d*. Refining the structure with framework cations and O atoms results in only two strong residual electron-density peaks at the 24*d* and 96*h* positions. Assuming that Li atoms occupy only these two positions, then there must be a distinct overpopulation at the 24*d* site. Thus, Al^III^ is assigned to the 24*d* site and its content was fixed using the chemical com­position calculated from energy dispersive X-ray (EDX) analysis on a similar material synthesized using an identical experimental setup (Rettenwander *et al.*, 2016 ▸). The Li content was allowed to refine freely.

For pristine Ga-stabilized LLZO, indexing of the diffraction data yields the space-group symmetry *I* 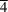 3*d*. Three different sites are identified from residual electron-density maps for the Li^I^ ions: at Wykoff positions 12*a*, 12*b* and 48*e*. It is evident that Ga^III^ must be located at the 12*a* position, as the 12*a* site becomes distinctly overpopulated when refined with only Li^I^. In subsequent refinements, both the Li1 and Li2 sites are assumed to be fully occupied and the electron density is modelled with Li + Ga = 1. The result is that Ga^III^ almost exclusively resides at the 12*a* position. Refinement of the anisotropic atomic displacement parameters (adps) is possible for all atoms using the same strategy as that applied by Wagner *et al*. (2016*a* ▸,*b* ▸). The data of the hydro­thermally treated Ga-LLZO sample can also be indexed using *I* 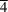 3*d* symmetry when the model for untreated material is used as the starting point. The Li1 site is again assumed to be fully occupied and its electron density was modelled with Li + Ga = 1. This approach is considered valid as the resultant Ga^III^ content is similar (albeit slightly higher) to that obtained for the untreated sample. The Li2 and, in particular, the Li3 sites are distinctly depleted in Li and no anisotropic atomic displacement refinement is possible. Thus, the isotropic adps are adjusted and fixed to the *U*_eq_ value refined for the Li1 site; anisotropic adps could be obtained for Li1. Protons are located close to the O1 atom using residual electron-density maps. Fixing the *U*_eq_ value of hydrogen yields reliable occupation factors for this site and an almost charge-balanced chemical formula (with a slight surplus of 0.35 negative charges).

For deeply hydrated Al-LLZO, indexing of data yields a change in symmetry from *Ia* 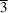 *d* to acentric *I* 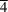 3*d*. The structure of this com­pound is refined using the model of the Ga-stabilized LLZO, as described above for the La-, Zr- and the two O-atom positions. The electron densities at Li1 and Li2 were modelled first with only Li^+^ ions. In this case, the Li1 position is distinctly overpopulated, while the occupation of the Li2 position is low, and there is no indication that the Li3 position is occupied at all. Consequently, all Al^III^ is assigned to the Li1 site and its occupation is fixed to the value used in the unaltered sample, consistent with the assumption that no Al left the structure during hydration. Refinement of the anisotropic adps is not possible for the Li1 site, and the isotropic adp is very small, so the isotropic adp of Li1 was adjusted in such a way that it has a similar value to the *U*_eq_ value of the Li2 site (where anisotropic adp refinements were possible) and fixed as such in subsequent refinements. Two distinct residual electron-density peaks are identified in residual electron-density maps: one high (2.5 e Å^−3^), very close to the Zr-atom position, and another at ∼0.8 Å from the O1 atom. This latter (*x*, *y*, *z*) position is close to the proposed H-atom positions given by Larraz *et al.* (2013 ▸) and Orera *et al.* (2016 ▸). Hence, this residual density is assigned to the H atom, which is bonded to the O1 atom. Independent refinement of the *x*, *y* and *z* positions, and the occupation of the H atom is possible, whereas the isotropic adp had to be fixed. A residual density close to Zr can be explained by positional disorder at this site, similar to that reported for deeply hydrated Ta-substituted LLZO, which also transformed to the *I* 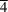 3*d* structure. How­ever, there is a marked decrease in the reliability factors associated with the refinements when Zr^IV^ disorder is applied, *e.g.* there is no sign of another site close to O2 that would allow for another H atom to be bonded to the O2 atom. Furthermore, no reliable residual electron-density peaks can be detected.

Two additional crystals were hydrated in the same way and both were then analysed; the results are consistent with those reported in the tables and below.

## Results and discussion

**Figure 1:**
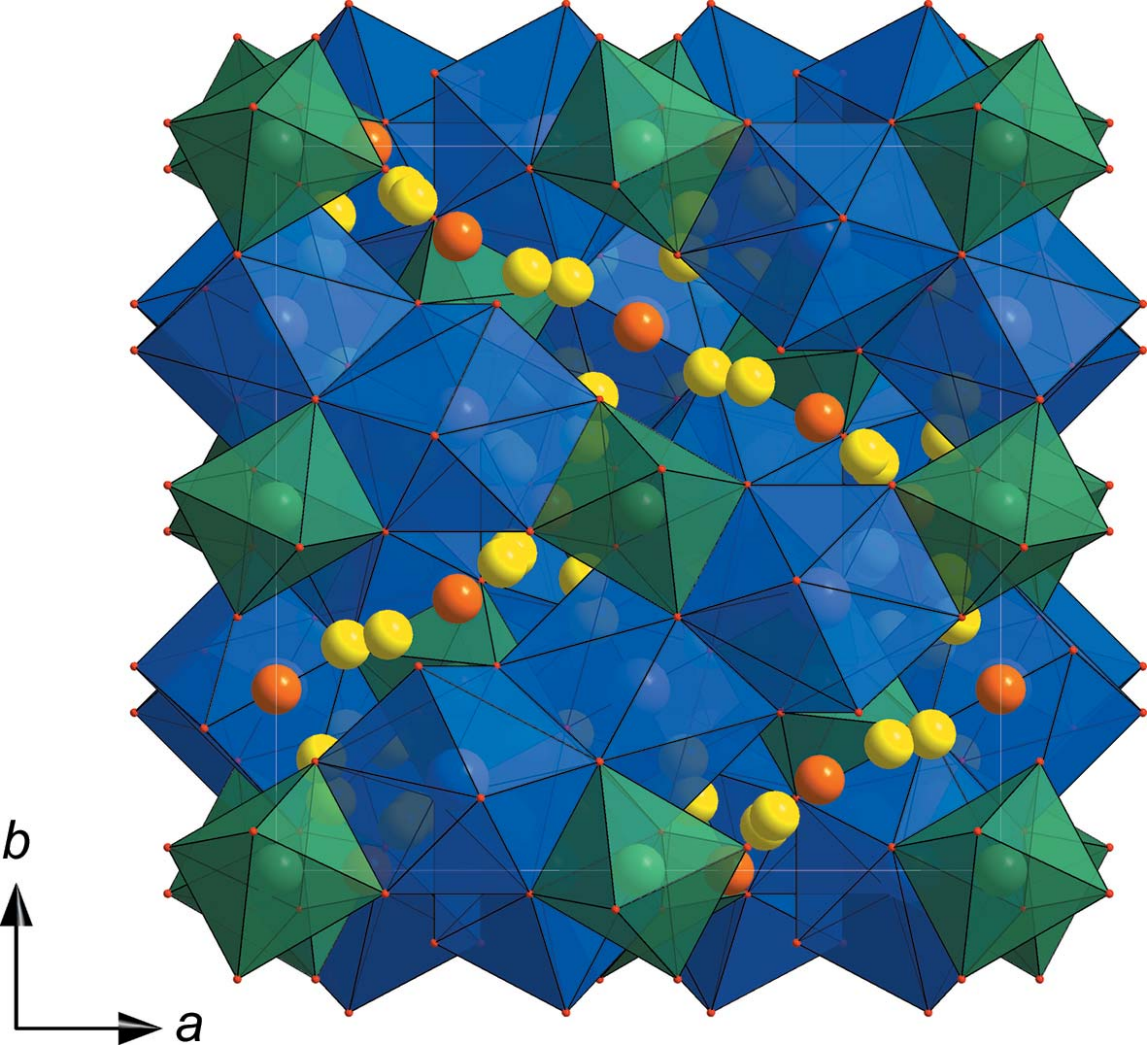
The crystal structure of Al-stabilized LLZO (space group *Ia* 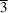 *d*) in a polyhedral format. LaO_8_ sites are in light blue and ZrO_6_ octa­hedra in sea green, whereas the Li1 (orange) and Li2 sites (yellow) are shown as spheres only for clarity and to highlight their diffusion pathway.

In the pristine state, Al-LLZO shows the typical garnet structure with *Ia* 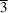 *d* symmetry. La^III^ occupies the eightfold-coordinated 24*c* site with two symmetrically independent La—O bond lengths (see Table 2 ▸). A slight deficit in the La^III^ ion site occupation is observed, which is in line with previous studies (Hiebl *et al.*, 2019 ▸; Rettenwander *et al.*, 2016 ▸; Wagner *et al.*, 2016*a* ▸,*b* ▸). The Zr^IV^ ions are located at the 16*a* position with a regular sixfold oxygen coordination and a bond length of 2.076 (16) Å. As depicted in Fig. 1 ▸, the garnet structure com­prises an integrated framework constructed of edge-sharing octa­hedral and dodeca­hedral sites, in which the Li atoms are located at both the regular 24*d* tetra­hedral site (Li1) and at the inter­stitial 96*h* position (Li2), often denoted to have a distorted octa­hedral coordination [Li2—O distances range between 1.854 (15) and 2.646 (14) Å, with the average of the four smaller bond lengths being 2.085 Å]. Al^III^ substitutes into the 24*d* position and the four equivalent Li—O lengths are 1.9044 (17) Å; both the 24*d* and the 96*h* positions show distinct vacancies.

**Figure 2:**
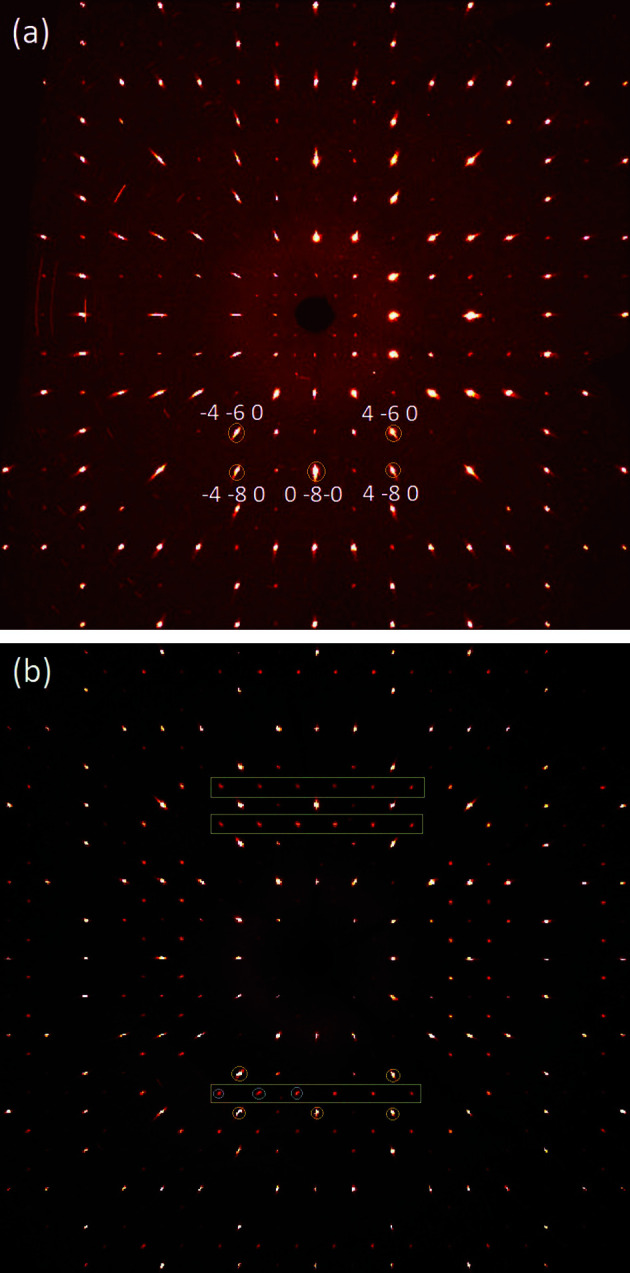
Reconstructed precession images of the *hk*0 plane of (*a*) a single crystal of Al-stabilized LLZO directly after synthesis with some selected Bragg reflections indexed and (*b*) a single crystal after hydro­thermal alteration at 150 °C for 28 d. Note the presence of sharp superstructure reflections, which obey *Ia* 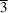 *d* symmetry. In part (*b*), the yellow encircled Bragg reflections are the same as in part (*a*), while the blue encircled reflections in the rectangular box correspond (from left to right) to the 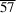 0, 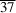 0, 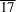 0,… Bragg reflections.

The hydro­thermally altered sample of Al-LLZO shows additional Bragg peaks of type *k* = odd and *l* = odd that obey *Ia* 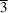 *d* symmetry. Calculated precession images of the *hk*0 plane for unaltered and altered Al-LLZO are com­pared in Fig. 2 ▸, where some Bragg peaks that obey *Ia* 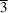 *d* symmetry are marked. Indexing of the observed data definitively yield the space-group symmetry *I* 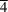 3*d*, in accordance with the findings of Larraz *et al.* (2013 ▸) and Orera *et al.* (2016 ▸) for Li^I^/H^I^-exchanged LLZO. The symmetry reduction is associated with several rearrangements in the structural architecture. The oxygen site at 96*h* in *Ia* 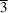 *d* splits into two different 48*e* positions, *i.e.* O1 and O2, in *I* 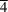 3*d*, thus allowing for two different sets of Zr—O and four different La—O bond lengths. The Zr^IV^ ion shifts from (0, 0, 0) with site symmetry 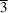 (*Ia* 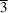 *d*) to the 16*c* position (*x*, *x*, *x*) with site symmetry 3 (*I* 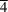 3*d*). Some positional disorder is observed at the Zr position, where around 20% of the Zr^IV^ is displaced to a general 48*e* position, with a Zr—Zr offset of ∼0.39 (3) Å. Free refinement of the site occupancies of these two Zr positions total 1.96 Zr^IV^ atoms per formula unit, *i.e.* very close to the expected value of 2.0. Whereas in *Ia* 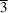 *d*, the six Zr—O bond lengths are equivalent to a value of 2.1076 (16) Å. In the hydro­thermally altered structure, Zr^IV^ is in a very distorted octa­hedral environment at 16*c*, with Zr1*A*—O1^xi^ bond lengths of 2.007 (4) Å (×3), and Zr1*A*—O2^xii^ of 2.217 (4) Å (×3) (see Table 2 ▸ for symmetry codes). For the general position, the Zr1*B*—O bonds range between 1.85 (3) and 2.36 (3) Å (Table 2 ▸). It is inter­esting to note that a similar behaviour was found during hydro­thermal alteration of Li_6_La_3_ZrTaO_12_ (LLZTO). Pristine LLZTO shows *Ia* 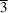 *d* space-group symmetry but deep hydration induces a symmetry reduction to *I* 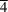 3*d*. A disorder at the Zr/Ta site is also observed in hydrated LLZTO similar to that in hydrated Al-stabilized LLZO in this study. It would appear that the symmetry reduction, induced by Li^I^/H^I^ exchange, causes a large distortion of the O-atom environment around the 16*c* position and induces some positional disorder.

The regular 24*d* tetra­hedral site of the *Ia* 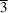 *d* garnet structure splits into two different sites, 12*a* (Li1) and 12*b* (Li2), upon symmetry reduction to *I* 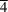 3*d*. Al^III^ is ordered onto the 12*a* (Li1) site but a distinct number of vacancies are observed on both sites. While ∼4.5 apfu Li^I^ occupy the inter­stitial 96*h* position in the pristine state, this position (Li3 at 48*e*) is com­pletely unoccupied in the deeply hydrated form, *i.e.* all the Li^I^ ions have vacated the inter­stitial octa­hedral site.

**Figure 3:**
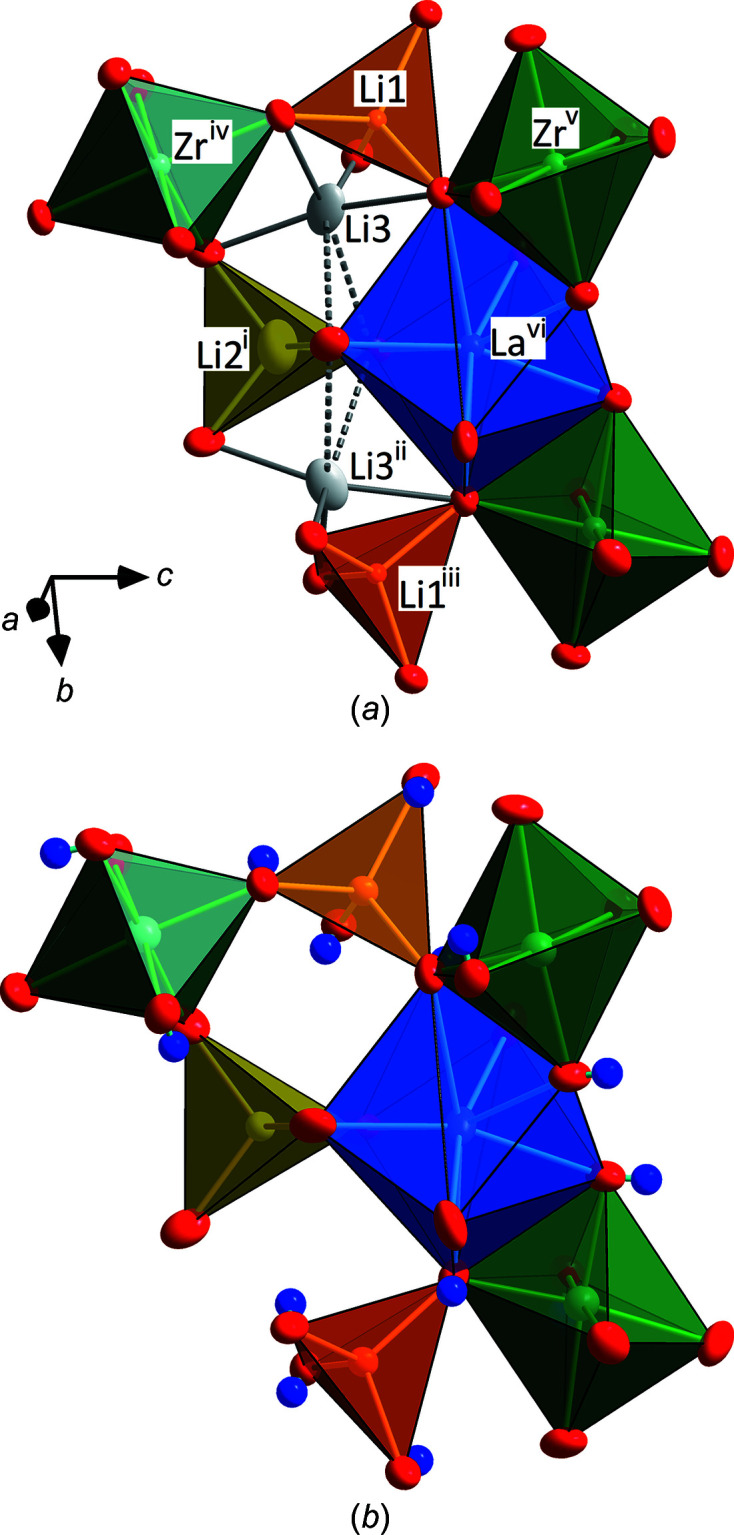
Polyhedral representations of part of the crystal structure of Ga-sta­bil­ized LLZO (space group *I* 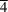 3*d*) in the (*a*) pristine and (*b*) hydro­ther­mally altered state viewed on the (12, 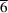 ,3) plane. LaO_8_ sites are in light blue, ZrO_6_ octa­hedra in green and Li1, Li2 and Li3 sites in orange, yellow and grey, respectively. The dashed grey bonds around the Li3 site are the two most distant. [Symmetry codes: (i) −*z* + 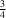 , −*y* + 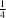 , *x* − 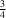 ; (ii) −*z* + 1, −*x* + 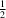 , *y*; (iii) −*x* + 1, −*y* + 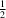 , *z*; (iv) −*x* + 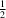 , *y*, −*z*; (v) −*x* + 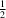 , −*y*, *z* + 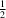 ; (vi) −*z* + 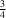 , −*y* + 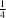 , *x* + 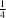 .]

Recently, Redhammer *et al.* (2021*b* ▸) observed a progressive increase of the tetra­hedral site (24*d* and 12*a*) occupation in Al-stabilized LLZO during continuous Li^I^/H^I^ exchange in a humid atmosphere and under mild hydro­thermal conditions. The shift and ordering of Li^I^ from the inter­stitial site to the regular tetra­hedral site, and the preference of Li^I^ and Al^III^ for the 12*a* position, are described as triggers for the symmetry reduction from *Ia* 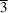 *d* to *I* 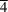 3*d* (Redhammer *et al.*, 2021*b* ▸). A study of the structure of the deeply hydrated samples here shows that, after com­plete recovery of Li^I^ from the inter­stitial site, the tetra­hedral 12*a* and 12*b* sites also take part in the Li^I^/H^I^ exchange. When com­pared to the data of Redhammer *et al.* (2021*b* ▸), it is obvious that, in this study, more Li^I^ is extracted from the 12*a* site [∼1.4 to 0.64 (2) apfu], but there is only a moderate change in site occupation at 12*b* [∼1.1 to 0.82 (2) apfu]. As Li^I^ is extracted from the structure, H^I^ is incorporated and bonds with the O1 atom. The Li^I^ ions in the 12*a* tetra­hedron are thus coordinated by four OH groups, with the O—H vector pointing towards the empty Li3 site (com­pare with Fig. 3 ▸). The proposed position of H^I^ is in line with that found by Orera *et al.* (2016 ▸) for pure undoped LLZO based on neutron diffraction on polycrystalline powders. Refinements indicate that the deeply hydrated Al-LLZO has a com­position of La_2.88_Zr_1.95_Al_0.15_Li_1.64_H_5.52_O_12_. It is also worth noting that a significantly lower number of La^III^ ions are found at the 24*d* site, so it would appear that La^III^ also leaves the structure under hydro­thermal conditions. This is in line with the observations of Redhammer *et al.* (2021*a* ▸,*b* ▸), who observed an instability of LLZO powders in highly humid air, with decom­position of LLZO, leading to the formation of lanthanite La_2_(CO_3_)_3_·8H_2_O within ∼30 d of exposure.

The Li^I^/H^I^ exchange is accom­panied by a large increase in the *a* unit-cell parameter from 12.9637 (2) to 13.0738 (2) Å, which is among the largest values yet recorded for hydrated LLZO, *cf.* 13.06245 (4) Å at 77 K for hydrated LLZO with an Li_2.3_H_4.7_La_3_Zr_2_O_12_ com­position (Orera *et al.*, 2016 ▸) or 13.0530 (8) Å for Li_3.08_H_3.52_La_3_Zr_2_O_12_Ta_0.4_ (Yow *et al.*, 2016 ▸). A replacement of stronger Li—O bonds by weaker O—H bonds and the creation of a large number of vacant sites, especially around the empty 48*e* (Li3) site, both require more space and are considered responsible for the lattice expansion.

Pristine unaltered Ga-stabilized LLZO shows *I* 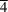 3*d* sym­metry. A section of this crystal structure is illustrated in Fig. 3 ▸, together with that of the hydro­thermally treated sam­ple. Both the tetra­hedrally coordinated 12*a* and 12*b* positions appear to be fully occupied, with the Ga^III^ ions ordered onto the 12*a* position. In contrast to the Li-stuffed garnets with *Ia* 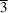 *d* symmetry, the decreased number of vacancies at the regular tetra­hedral sites are considered to be a characteristic feature of the *I* 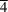 3*d* garnet structure. The 48*e* inter­stitial site [Li3 in Fig. 3 ▸(*a*)] is occupied by 3.71 Li^I^ apfu, equivalent to being ∼62% full. All of the tetra­hedral faces of the fourfold oxygen coordination around the Li1 and Li2 sites are shared with neighbouring Li3 sites, with inter­atomic contacts of 1.645 (8) (Li1—Li3) and 2.340 (9) Å (Li2—Li3), thereby forming a three-dimensional network that is responsible for the good Li-ion conductivity in this com­pound. The dodeca­hedral site has a small number of vacancies, while the regular octa­hedral positions are fully occupied with Zr^IV^, resulting in a com­position of La_2.94_Zr_2.00_Ga_0.28_Li_6.43_O_12_ for the unaltered Ga-stabilized LLZO material.

A smaller and larger set of La—O1/O2 bonds are observed at the dodeca­hedral site [2.4935 (19)–2.5264 (19) and 2.587 (2)–2.5975 (19) Å] and the octa­hedron is regular with two inde­pen­dent Zr—O bonds ranging between 2.0823 (18) and 2.1346 (18) Å. Therefore, the octa­hedra are much less distorted than those in the altered Al-LLZO with the same symmetry (Table 2 ▸). The Li1 (12*a*) site, which hosts Ga^III^, is slightly smaller than the Li2 (12*b*) site, whereas the Li3 site, as in Al-LLZO, shows a very distorted 4 + 2-fold coordination, with bond lengths between 1.878 (4) and 2.639 (8) Å; the average of the four shorter bonds is 2.073 Å, *i.e.* slightly smaller than in the pristine Al-LLZO [see Fig. 3 ▸(*a*)].

As in Al-LLZO, hydro­thermal treatment of Ga-LLZO induces Li^I^/H^I^ exchange. Again, a large increase of the lattice parameter to 13.06720 (12) Å is close to the value in hydrated Al-LLZO, suggesting that values around 13.07 Å represent an upper limit for lattice-parameter increase due to hydration. From site-occupation refinements, very minor Li^I^ ions are found at the inter­stitial 48*e* position, *i.e.* this site is almost com­pletely depleted. One key difference with Al-LLZO is that no Li^I^ is lost from the 12*a* position. It seems that Ga^III^ ions pin Li^I^ at this site. However, Li^I^ ions are extracted from the 12*b* site where the amount of Li^I^ is reduced from 1.49 apfu in the pristine to 0.50 apfu in the altered sample. Protons are again located close to the O1 atom [Fig. 3 ▸(*b*)], giving rise to a fully hydrated Li1(OH)_4_ coordination around the 12*a* site. Some differences to Al-LLZO are, however, evident: there is no significant reduction in the La-site occupation and no residual electron density is observed close to the Zr^IV^ ions, *i.e.* there is no indication of disorder at the 16*c* position in altered Ga-LLZO. Nevertheless, the ZrO_6_ octa­hedron is much more distorted in the altered sample, with Zr—O bond lengths ranging between 2.03 (3) and 2.187 (4) Å; the difference between the two independent Zr—O bonds increases with pro­longed Li^I^/H^I^ exchange. This was outlined by Redhammer *et al.* (2021*b* ▸) and the data of this study fit the extrapolated trends observed there. A distinct alteration is also observed within the coordination sphere of the 24*d* site due to H^I^ incorporation. The most prominent effects include the reduction of the longer La1—O1^xvii^ distance by ∼0.073 Å, as well as changes in the three other La—O bond lengths. In addition, Li1—O1^xv^ and Li2—O2^xvi^ are extended in the altered sample (see Table 2 ▸).

## Supplementary Material

Crystal structure: contains datablock(s) global, LLZO-Al15-pristine, LLZO-Al15-hydro-150C, LLZO-Ga40-pristine, LLZO-Ga40-hydro-150C. DOI: 10.1107/S2053229621012250/ep3020sup1.cif

Structure factors: contains datablock(s) LLZO-Al15-pristine. DOI: 10.1107/S2053229621012250/ep3020LLZO-Al15-pristinesup2.hkl

Structure factors: contains datablock(s) LLZO-Al15-hydro-150C. DOI: 10.1107/S2053229621012250/ep3020LLZO-Al15-hydro-150Csup3.hkl

Structure factors: contains datablock(s) LLZO-Ga40-pristine. DOI: 10.1107/S2053229621012250/ep3020LLZO-Ga40-pristinesup4.hkl

Structure factors: contains datablock(s) LLZO-Ga40-hydro-150C. DOI: 10.1107/S2053229621012250/ep3020LLZO-Ga40-hydro-150Csup5.hkl

CCDC references: 2122845, 2122844, 2122843, 2122842

## Figures and Tables

**Table 1:** Experimental details For all structures: *Z* = 8. Experiments were carried out at 298 K with Mo *K*α radiation using a Bruker SMART APEX diffractometer. Absorption was corrected for by multi-scan methods (*APEX2*; Bruker, 2012 ▸). **LLZO-Al15-pristine** = fresh sample of Al-doped LLZO, measured directly after the end of the synthesis, **LLZO-Al15-hydro-150C** = Al-doped LLZO aged hydrothermally at 150 °C, **LLZO-Ga40-pristine** = fresh sample of Ga-doped LLZO, measured directly after the end of the synthesis and **LLZO-Ga40-hydro-150C** = Ga-doped LLZO aged hydrothermally at 150 °C.

	**LLZO-Al15-pristine**	**LLZO-Al15-hydro-150C**	**LLZO-Ga40-pristine**	**LLZO-Ga40-hydro-150C**
Crystal data				
Chemical formula	Al_0.15_La_2.95_Li_5.73_O_12_Zr_2_	Al_0.15_H_5.52_La_2.88_Li_1.64_O_12_Zr_1.95_	Ga_0.28_La_2.94_Li_6.44_O_12.00_Zr_2.00_	Ga_0.26_H_3.90_La_2.96_Li_1.99_O_12_Zr_2_
*M*_r_	827.89	790.95	847.67	821.46
Crystal system, space group	Cubic, *I* *a*\overline{3}*d*	Cubic, *I*\overline{4}3*d*	Cubic, *I*\overline{4}3*d*	Cubic, *I*\overline{4}3*d*
*a* (Å)	12.9637 (2)	13.0738 (2)	12.9669 (2)	13.06720 (12)
*V* (Å^3^)	2178.65 (10)	2234.63 (10)	2180.26 (10)	2231.25 (6)
μ (mm^−1^)	13.24	12.61	13.88	13.57
Crystal size (mm)	0.12 × 0.11 × 0.07	0.13 × 0.12 × 0.08	0.13 × 0.13 × 0.10	0.12 × 0.11 × 0.09

Data collection				
*T*_min_, *T*_max_	0.22, 0.39	0.21, 0.36	0.19, 0.25	0.21, 0.36
No. of measured, independent and observed [*I* > 2σ(*I*)] reflections	33130, 455, 441	34201, 819, 819	35033, 1054, 1045	35657, 918, 915
*R*_int_	0.029	0.026	0.038	0.028
(sin θ/λ)_max_ (Å^−1^)	0.840	0.804	0.884	0.838

Refinement				
*R*[*F* ^2^ > 2σ(*F* ^2^)], *wR*(*F* ^2^), *S*	0.016, 0.032, 1.51	0.013, 0.029, 1.27	0.013, 0.026, 1.29	0.018, 0.037, 1.12
No. of reflections	455	819	1054	918
No. of parameters	25	44	48	43
No. of restraints	0	1	2	3
H-atom treatment	–	Only H-atom coordinates refined	–	Only H-atom coordinates refined
Δρ_max_, Δρ_min_ (e Å^−3^)	0.71, −0.44	0.58, −0.51	0.53, −0.65	0.54, −0.52
Absolute structure	–	Refined as an inversion twin	Refined as an inversion twin	Refined as an inversion twin
Absolute structure parameter	–	0.50 (3)	0.50 (4)	0.99944 (15)

Computer programs: *APEX2* (Bruker, 2012 ▸), *SIR2014* (Burla *et al.*, 2012 ▸), *SHELXL2014* (Sheldrick, 2015 ▸), *ORTEP-3 for Windows* (Farrugia, 2012 ▸) and *WinGX* (Farrugia, 2012 ▸).

**Table 2:** Selected geometric parameters (Å)

**LLZO-Al15-pristine**			
La1—O1^i^	2.5110 (17)	Li2—O1	1.854 (15)
La1—O1	2.5951 (17)	Li2—O1^vii^	2.085 (14)
Zr1—O1^ii^	2.1076 (16)	Li2—O1^vi^	2.159 (15)
Li1—Li2^iii^	1.616 (14)	Li2—O1^viii^	2.242 (14)
Li1—O1^iv^	1.9044 (17)	Li2—Li2^viii^	2.46 (2)
Li1—Li2^v^	2.367 (14)	Li2—O1^ix^	2.646 (14)
Li2—Li2^vi^	0.79 (3)		
			
**LLZO-Al15-hydro-150C**			
La1—O1^i^	2.506 (3)	Zr1*B*—O2^xii^	2.06 (3)
La1—O1	2.518 (3)	Zr1*B*—O2^iv^	2.10 (3)
La1—O2^x^	2.544 (3)	Zr1*B*—O1^ii^	2.14 (3)
La1—O2^i^	2.607 (3)	Zr1*B*—O1^xiv^	2.19 (3)
Zr1*A*—O1^xi^	2.007 (4)	Zr1*B*—O1^xi^	2.36 (3)
Zr1*A*—O2^xii^	2.217 (4)	Li1—O1^xv^	1.998 (3)
Zr1*B*—O2^xiii^	1.85 (3)	Li2—O2^xvi^	1.976 (3)
			
**LLZO-Ga40-pristine**			
La1—O2^xiii^	2.4935 (19)	Li2—O2^xvi^	1.9246 (19)
La1—O1^i^	2.5264 (19)	Li2—Li3^xviii^	2.340 (9)
La1—O2^i^	2.587 (2)	Li3—O1	1.878 (14)
La1—O1^xvii^	2.5975 (19)	Li3—O1^vii^	2.075 (8)
Zr1—O2^xiii^	2.0823 (18)	Li3—O2^i^	2.108 (9)
Zr1—O1^ii^	2.1346 (18)	Li3—O1^viii^	2.229 (8)
Li1—Li3^iii^	1.645 (8)	Li3—Li3^vii^	2.513 (13)
Li1—O1^xv^	1.8941 (18)	Li3—O2^vii^	2.639 (8)
			
**LLZO-Ga40-hydro-150C**			
La1—O1^i^	2.514 (3)	Li1—Li3^iii^	1.5 (2)
La1—O1^xvii^	2.525 (3)	Li1—O1^xv^	1.987 (4)
La1—O2^xiii^	2.539 (4)	Li2—O2^xvi^	1.982 (4)
La1—O2^i^	2.603 (4)	Li2—Li3^xviii^	3.2 (2)
Zr1—O1^ii^	2.031 (3)	Li3—O1^vii^	2.4 (2)
Zr1—O2^xiii^	2.187 (4)	Li3—Li3^viii^	2.6 (4)

Symmetry codes: (i) *z*, *x*, *y*; (ii) *x* − {1\over 4}, *z* − {1\over 4}, *y* − {1\over 4}; (iii) −*z* + {3\over 4}, *y* − {1\over 4}, −*x* + {1\over 4}; (iv) *z*, −*x*, −*y* + {1\over 2}; (v) −*y* + {1\over 2}, *z* − {1\over 2}, *x*; (vi) −*x* + {1\over 4}, *z* − {1\over 4}, *y* + {1\over 4}; (vii) *y* − {1\over 4}, −*x* + {1\over 4}, −*z* + {3\over 4}; (viii) −*y* + {1\over 4}, *x* + {1\over 4}, −*z* + {3\over 4}; (ix) *y*, −*z* + {1\over 2}, *x* + {1\over 2}; (x) −*x*, *y* − {1\over 2}, −*z* + {1\over 2}; (xi) *z* − {1\over 4}, *y* − {1\over 4}, *x* − {1\over 4}; (xii) −*y* + {1\over 2}, *z*, −*x*; (xiii) −*x*, −*y* + {1\over 2}, *z*; (xiv) *y* − {1\over 4}, *x* − {1\over 4}, *z* − {1\over 4}; (xv) −*z* + {3\over 4}, −*y* + {1\over 4}, *x* + {1\over 4}; (xvi) *x* + {3\over 4}, −*z* + {1\over 4}, −*y* + {3\over 4}; (xvii) *x*, −*y*, −*z* + {1\over 2}; (xviii) *y* + {3\over 4}, −*x* + {1\over 4}, −*z* + {3\over 4}.
